# Bioleaching of GaAs metal hydroxide sludge: a biohydrometallurgical alternative to conventional acid leaching

**DOI:** 10.3389/fmicb.2026.1807986

**Published:** 2026-06-10

**Authors:** Mareike Thea Fritze, Frank Haubrich, Anna Otto, Axel Schippers, Sabrina Hedrich

**Affiliations:** 1 Department of Biosciences, TU Bergakademie Freiberg, Freiberg, Germany; 2 G.E.O.S. Ingenieurgesellschaft mbH, Halsbrücke, Germany; 3 Federal Institute for Geosciences and Natural Resources (BGR), Hannover, Germany

**Keywords:** acidophiles, bioleaching, gallium, industrial waste, iron reduction, recycling

## Abstract

The economically important metal gallium (Ga) is considered to be a critical raw material with a potentially high supply risk. The industrial semiconductor production process generates multiple waste streams that contain Ga in economically significant amounts. This study explores bioleaching for Ga recycling from a waste metal hydroxide sludge originating from industrial GaAs-wafer production. Previous studies have demonstrated that hydrometallurgical recycling of this sludge provides an effective approach for gallium recovery. Furthermore, it was shown that reducing the ferric iron in the leaching solution has a beneficial effect on downstream processing, enabling less consumption of chemicals. Based on these results, the present study investigates the potential of bioleaching using *Acidithiobacillus thiooxidans* with the addition of elemental sulfur for the recovery of gallium and the reduction of ferric iron from the metal hydroxide sludge. Shake flask experiments showed that 60% of the total iron in the leachate was present as ferrous iron after processing 3% (w/v) sludge after 8 days. Subsequent reactor experiments at 3% (w/v) solid load resulted in only 30% iron reduction after 14 days, while element recoveries reached 80% for Fe, 100% for Ga, and 99% for As. A further bioreactor experiment with a gradual addition of the sludge up to 5% (w/v) led to a 70% reduction of ferric iron after 14 days. The experiments revealed that microbially mediated ferric iron reduction decreased at pH values below 1.0. Fluoride was found to inhibit microbial activity during the experiments, which was mitigated by the addition of aluminum at an Al:Fe ratio of 1.4:1. Overall, this study demonstrates that bioleaching is a possible approach for the recycling of gallium-containing metal hydroxide sludge.

## 1 Introduction

Every year significant amounts of metal-containing residues are produced by various industries, e.g., metallurgical sludges, slags, dusts, ashes, and tailings (Spooren et al., 2020), as well as electronic waste and spent catalysts (Kinnunen and Hedrich, 2023). These waste streams have the potential to serve as secondary raw materials, as they can be recycled or processed for metal recovery (Blengini et al., 2020; Kinnunen and Hedrich, 2023). The semiconductor industry generates multiple waste streams, including metal-rich sludges that contain significant concentrations of metals, such as gallium (Ga) (Ueberschaar et al., 2017). Especially gallium possesses a supply risk because of its low natural abundance and since its extraction from minerals is challenging (Oliveira et al., 2021). Ga occurs in small quantities in aluminum or zinc ores (Oliveira et al., 2021; Font et al., 2007) or in industrial residues such as red mud or coal fly ash (Lu et al., 2017; Oliveira et al., 2021) and Ga-rich wastes from the electronics industry (Swain et al., 2015a,b; Lu et al., 2017). Gallium (Ga) is mainly produced in China (Deutsche Rohstoffagentur, 2025), while it is also obtained in Canada and Germany through recycling processes (Oliveira et al., 2021). Gallium is used as gallium arsenide (GaAs) or gallium nitride (GaN) in modern semiconductors and LED chips, which are incorporated in many electronic devices such as cell phones, photovoltaic systems, optical communication devices and computers (Lu et al., 2017; Castro et al., 2024). The high demand for this element has led to a focus on developing efficient recovery processes from secondary resources (Castro et al., 2024). During production of GaAs semiconductor chips, most of the initially used gallium is lost in the course of production processes as waste material (Ueberschaar et al., 2017; Haubrich et al., 2020). The former German EcoGaIN research project aimed to develop innovative processes for the sustainable recovery of gallium from waste streams generated during GaAs semiconductor production and to reuse it in the manufacturing cycle (Haubrich et al., 2020). One type of waste material is metal hydroxide sludge, generated in the neutralization plant of various production waters. In addition to iron hydroxide, this sludge also contains Ga and toxic elements such as As. In the course of the EcoGaIN project, different chemical leaching processes were examined to extract gallium from the sludge. The results showed that the reduction of ferric iron in the leach solution lowers the demand for chemicals during downstream processing (Haubrich et al., 2020). Besides the hydrometallurgical recycling route for metal hydroxide sludge studied in the EcoGaIN project, biohydrometallurgical approaches might be another viable option.

Biohydrometallurgy comprises methods to recover metals from the liquid phase or extracting metals from solids (bioleaching) using various biological processes (Vera et al., 2022; Hedrich et al., 2023). For bioleaching, acidophilic bacteria and archaea are widely used, as they grow optimally at pH values below 3.0 and produce lixiviants, such as acids, through their metabolic activities (Vera et al., 2022). Commercially, bioleaching is used for the recovery of metals like copper, zinc, nickel and cobalt from ores (Roberto and Schippers, 2022) and metallurgical side streams (Gericke et al., 2023). The application of bioleaching for waste materials such as electronic scrap, catalysts, sludges or slags has been tested at laboratory scale (Bosecker, 1987; Srichandan et al., 2019; Kinnunen and Hedrich, 2023). Biohydrometallurgical recycling of Ga-containing materials is already described in the literature (Maneesuwannarat et al., 2016; Wang et al., 2018; Pourhossein and Mousavi, 2018, 2019; Schippers et al., 2019). For instance, the use of bioleaching to recover valuable metals from light-emitting diode waste was investigated by using microbially-produced ferric iron as a leaching agent to recover 84% Ga at 2% solid load (Pourhossein and Mousavi, 2019). Additionally, almost 100% Ga was selectively recovered from aluminum smelter slag via bioleaching at 2% solid (Wang et al., 2018). Bioleaching of crushed and screened sphalerite-rich ore achieved extraction of up to 1 mg/L gallium as a trace element (Schippers et al., 2019). Although the recovery of Ga via bioleaching is usually high, there are still challenges such as the alkaline nature of mainly Ga-containing materials, which leads to high acid consumption. Also, the high complexity of the materials, organic compounds (e.g., from plastics, resins), fluoride or bromine can be problematic. Furthermore, many waste materials consist of metal oxides requiring a different bioleaching approach with addition of substrates such as elemental sulfur or organic carbon sources, than that for metal sulfides (Kinnunen and Hedrich, 2023). There are three principal bioleaching mechanisms: acid, oxidative, and reductive bioleaching (Vera et al., 2022). Besides acid leaching, reductive bioleaching is of particular significance in this study. It includes the dissolution of solids by chemical reduction, catalyzed by microorganisms (Vera et al., 2022). The process typically relies on the reduction of ferric iron, a reaction that can occur under anaerobic, micro-aerobic and aerobic conditions (Malik and Hedrich, 2022).

The phylogeny of ferric iron-reducing acidophiles is very diverse, including chemoorganotrophic (organic matter as electron donor) and chemolithotrophic species (H₂ or S^0^ as electron donor). So far, most studies focused on dissimilatory ferric iron reduction coupled to oxidation of reduced inorganic sulfur compounds (DIRSO) under anaerobic conditions by *Acidithiobacillus* (*At.*) *ferrooxidans* (Breuker and Schippers, 2024), as summarized by Malik and Hedrich (2022). In addition, ferric iron reduction coupled to sulfur oxidation has been observed for various *Acidithiobacillus* species under aerobic conditions as well (Marrero et al., 2015, 2017; Johnson et al., 2017; Smith and Johnson, 2018; Malik and Hedrich, 2022; Breuker and Schippers, 2024). Furthermore, cell-free supernatants (pH 1.0) of *At. caldus* and *At. ferridurans* cultivated on sulfur showed aerobic iron reduction (Johnson et al., 2017). It has been hypothesized that sulfur intermediates such as thiosulfate and hydrogen sulfide, produced during the enzymatic oxidation of the added elemental sulfur to sulfuric acid, chemically reduce ferric iron (see Equations 1, 2; Marrero et al., 2017; Marrero et al., 2015, 2017; Malik and Hedrich, 2022; Breuker and Schippers, 2024).


$$2\;{\mathrm{Fe}}^{3+}+{\mathrm{HS}}^{-}\to S^{{}^{\circ}}+H^{+}+2\;{\mathrm{Fe}}^{2+}$$ (1)

$$2\;{\mathrm{Fe}}^{3+}+2\;S_{2}{O_{3}}^{2-}\to S_{4}{O_{6}}^{2-}+2\;{\mathrm{Fe}}^{2+}$$ (2)

Breuker and Schippers (2024) investigated ferric iron reduction rates in various *Acidithiobacillus* strains, utilizing elemental sulfur or tetrathionate as electron donor. *Acidithiobacillus (A.) ferrooxidans*, capable of DIRSO under anaerobic conditions exhibited the highest reduction rates. Under aerobic conditions *A. thiooxidans* and *A. caldus* showed lower ferric iron reduction rates with the latter showing the lowest activity with sulfur as the electron donor. There was no pronounced pH dependence of ferric iron reduction rates in the range of pH 1.0 to 1.9 for the type strains of all three species but rates increased with increasing pH for four other *At. thiooxidans* strains. Johnson et al. (2017) previously described that the aerobic ferric iron reduction by *At. thiooxidans* only became significant at pH 1.2 and below. Thiosulfate was detected as intermediate during aerobic and anaerobic cultivation of *At. thiooxidans* and *At. ferrooxidans* with tetrathionate and ferric iron (Breuker and Schippers, 2024). Hydrogen sulfide was formed in sulfur-grown cultures under microaerobic conditions. The detected sulfur intermediates, released by the cells, support the hypothesis of the chemical ferric iron reduction by these intermediates (Marrero et al., 2020; Breuker and Schippers, 2024; Stanković and Schippers, 2024). Johnson et al. (2017) and Johnson and Pakostova (2021) showed that ferric iron reduction in aerobic cultures of *Acidithiobacillus* spp. might not only be coupled to sulfur oxidation, but also hydrogen oxidation. Reductive bioleaching is well studied for oxide ores such as limonitic ores, e.g., for the recovery of cobalt and nickel. Earlier studies mostly used *At. ferrooxidans* under anaerobic conditions, while more recent studies demonstrated that aerobic reductive bioleaching using a consortium of *At. thiooxidans* and *At. ferrooxidans* or cultures of various *At. thiooxidans* strains resulted in more efficient Co and Ni leaching compared to the anaerobic process with *At. ferrooxidans* alone (Hallberg et al., 2011; Nancucheo et al., 2014; Santos et al., 2020; Marrero et al., 2015, 2020; Roberto and Schippers, 2022; Santos and Schippers, 2023; Stanković et al., 2022; Hetz and Schippers, 2025). Aerobic reductive bioleaching has also been investigated in other processes, such as for manganese dioxide ore using an autotrophic mixed culture, which achieved 99% Mn extraction (Xin et al., 2015).

Based on the results of the hydrometallurgical recycling of metal hydroxide sludge in the EcoGaIN project, which demonstrated an effective approach for gallium recovery and that ferric iron reduction has a beneficial effect on the downstream process, this study aims to investigate the potential of aerobic reductive bioleaching for Ga recycling. Reductive bioleaching presents a promising approach as the biogenic sulfuric acid can effectively dissolve the sludge, while the indirectly (microbially-mediated) ferric iron reduction facilitates a less chemical-intensive downstream processing of the leach solutions. During the experiments, it was observed that the bacterial activity was inhibited by the release of fluoride from the solids, as previously reported by Fritze and Hedrich (2024). To enable an efficient bioleaching process, this inhibition was mitigated through aluminum addition. In the present study, the aluminum dosage was also systematically optimized.

## 2 Materials and methods

### 2.1 Characteristics of the metal-hydroxide sludge

Metal hydroxide sludge is a byproduct of the neutralization process during the industrial production of GaAs wafers. Table 1 shows the composition of the sludge analyzed by hand-held X-ray fluorescence spectroscopy (h-XRF, Bruker S1 Titan). Fluoride analysis was carried out by Eurofins Umwelt Ost GmbH (Germany) using ion-selective electrodes (ISE), which determined a concentration of 0.538% fluoride. Calcium in the sludge occurs as CaCO_3_ as well as CaF_2_. Iron is entirely present as ferric iron in form of amorphous ferrihydrite. Phosphorus results from surfactants and is presumably adsorbed as phosphate on the Fe-hydroxides. Arsenic is assumed to be associated with the iron phases as arsenate (As(V)), and gallium is also expected to be associated with the iron phase (Haubrich et al., 2020). For the bioleaching experiments the sludge was dried at 60 °C for 48 h to reduce the water content to a negligible level and then homogenized using a mortar.

**Table 1:** Results of h-XRF analysis of the metal hydroxide sludge.

Element	Concentration [%]
Si	32.81
Fe	6.97
Ga	1.21
As	1.34
P	0.27
Ca	1.83

### 2.2 Optimization of aluminium addition to avoid fluoride inhibition

To avoid inhibition of the microbial activity by fluoride, the addition of aluminum to the bioleaching process was investigated. An aluminum sulfate (Al_2_(SO_4_)_3_ · xH_2_O) solution as well as sodium fluoride (NaF) solution, pH 1.8, was used to evaluate various Al:F ratios for optimization of the process. The investigations were carried out with *At. thiooxidans*^T^ (DSM 14887) using 3% (w/v) metal hydroxide sludge. Cultivation was carried out in 100 mL shake flasks (25 mL working volume, 120 rpm) using minimal basal salt medium (HBS) (Nancucheo et al., 2016) containing in g/L: Na_2_SO_4_∙10H_2_O 7.5; (NH_4_)_2_SO_4_ 22.5; KCl 2.5; MgSO_4_∙7H_2_O 25; KH_2_PO_4_ 2.5; Ca(NO_3_)_2_·4H_2_O 0.7, supplemented with a trace element solution containing (g/L): ZnSO_4_·7 H_2_O 10; CuSO_4_·5 H_2_O 1.0; MnSO_4_·4 H_2_O 1.0; CoSO_4_·7H_2_O 1.0; Cr_2_(SO_4_)_3_·15 H_2_O 0.5; H_3_BO_3_ 0.6; Na_2_MoO_4_·2H_2_O 0.5; NiSO_4_·6 H_2_O 1.0; Na_2_SeO_4_·10 H_2_O 1.0; Na_2_WO_4_·2 H_2_O 0.1. The pH of the medium was adjusted to 1.8 using sulfuric acid and 1% (w/v) elemental sulfur was provided as the substrate. Cultivation was carried out at 30 °C, with Al:F ratios 1:1; 1; 1.2:1; 1.3:1 and 1.4:1 being evaluated. The experiments were conducted in triplicates, incorporating a simple approach with Al:F ratios 0:1 (with bacteria), a positive control (with bacteria, without sludge and aluminum) and a negative control (without bacteria, Al:F ratio of 1.4:1). The required aluminum concentrations were calculated from the fluoride content of the sludge. Over an 11-day cultivation period, liquid samples were taken from all assays and pH (Mettler Toledo, InLab® Semi-Micro), redox potential (Mettler Toledo, InLab® Redox Micro, versus Ag/AgCl electrode), and concentrations of ferrous iron, ferric iron and total iron were measured (Lovley and Phillips, 1987; Pascualreguera et al., 1997).

### 2.3 Bioleaching in shake flasks

For aerobic reductive bioleaching the sulfur-oxidizing bacterium *At. thiooxidans*^T^ was used. The cultures were adapted to 5% (w/v) metal hydroxide sludge (with aluminum addition). The bioleaching experiments were examined in HBS minimal salt media at pH 1.8 and 1% elemental sulfur as substrate. The shake flask experiments (total volume 50 mL) were carried out with 3% (w/v) and 5% (w/v) of metal hydroxide sludge (see Table 2). Growth medium, sludge, and the required amount of sulfuric acid (as indicated in Table 2) were added to adjust the start pH of 1.8. Subsequently, aluminum (Al:F ratio of 1.5:1) was introduced, followed by the bacterial inoculum (10% (w/v)). Additionally, control experiments without inoculum were conducted, including a negative control (pH ~ 1.8) and an abiotic control (adjusted to the pH of the biotic treatments). The experiment was carried out in triplicates for all biotic setups and incubated for eight to 11 days at 30 °C. Sampling was performed in regular intervals to monitor pH, redox potential, ferrous iron, ferric iron and total iron concentration. Dissolved elements (Fe, As, and Ga) were measured using Inductively Coupled Plasma Mass Spectrometry (ICP-MS) (Thermo Scientific, XSeries 2).

**Table 2:** Conditions of shake flasks bioleaching experiments using metal hydroxide sludge at 3 an 5% (w/v) solid load.

3% (w/v)	5% (w/v)
1x HBS medium	1x HBS medium
1% S^0^	1% S^0^
12.9 mM Al (1.5:1 Al:F)	21.5 mM Al (1.5:1 Al:F)
10% inoculum	10% inoculum
1 mL H_2_SO_4_	2.6 mL H_2_SO_4_

### 2.4 Bioleaching experiments in bioreactors

Based on the experiments in the shake flasks, aerobic reductive bioleaching was also tested at a larger scale. A pH- and temperature-controlled, 2L- stirred tank bioreactor (Labfors 5, Infors AG) was used with a liquid working volume of 1 L. Aeration with compressed air (2 nL/min) and CO_2_ (0.1 nL/min) was achieved through a bent glass tube, while pH was monitored *in situ* (Polilyte Plus PHI S8 225). The bioreactor experiments were carried out with 3 and 5% (w/v) solid content with an Al:F ratio of 1.4:1. For each of the individual tests, a pre-culture (100 mL) containing 3% or 5% (w/v) metal hydroxide sludge was grown in a shake flask. The bioreactor tests differed in the mode of sludge addition, either direct or stepwise. For the stepwise addition, 1% (w/v) of sludge was added initially, and further 1% (w/v) material was introduced once the pH reached approximately < 1.3, until the respective solid load had been added completely. Table 3 presents an overview of the bioreactor tests with the respective parameters. Sampling was carried out at regular intervals to measure pH, redox potential and iron concentrations. Dissolved elements (Fe, As, and Ga) in the leaching solution from bioreactor tests with a 3% (w/v) solid load were analyzed using inductively coupled plasma optical emission spectrometry (ICP-OES; SPECTRO ARCOS FHX3X).

**Table 3:** Parameters of bioreactor tests for aerobic reductive bioleaching of metal hydroxide sludge.

Bioreactor experiment	Test 1	Test 2	Test 3
Solid load (w/v)	3%	3%	5%
Solid addition	Stepwise	Directly	Stepwise
Stirring speed [rpm]	450		500
Volume compressed air + CO_2_	compressed air 2 nL/min + 100 nmL/min CO_2_		compressed air 1 nL/min + 20 nmL/min CO_2_
Time [d]	14		14
Volume 2M H_2_SO_4_n [mL]	–	27.5	–

## 3 Results and discussion

### 3.1 Optimization of aluminum addition

An inhibition of the microbial activity was observed during preliminary growth experiments with the acidophilic sulfur-oxidizing bacterium *At. thiooxidans* in the presence of metal hydroxide sludge. This was due to the high fluoride content of the sludge, which is released in the medium during the bioleaching process (Fritze and Hedrich, 2024, 2026). Fluoride inhibition of microbial activity is strongly pH-dependent and is, e.g., minimal at pH ≥ 7.0, moderate at pH ~ 4.5, and strong at pH < 2.3 (Brierley and Kuhn, 2010; Suzuki et al., 1999). Therefore, the low pH conditions during bioleaching enhance the inhibitory effect of fluoride on microbial activity (Fritze and Hedrich, 2026). Under acidic conditions, fluoride exists partly as uncharged hydrogen fluoride (HF). Due to its high permeability HF readily crosses the cell membrane (Gutknecht and Walter, 1981). Inside the cell, HF dissociates into H^+^ and F^−^, lowering intracellular pH and inhibits enzymatic functions, ultimately inhibiting metabolism or causing cell death (Sicupira et al., 2011; Veloso et al., 2012). The fluoride-induced inhibition could be prevented by the addition of aluminum. By forming stable complexes with fluoride ions, that cannot pass the cell membrane, aluminum decreases the concentration of free fluoride and HF in the medium (Brierley and Kuhn, 2010; Sicupira et al., 2011; Veloso et al., 2012). Sicupira et al. (2011) reported that a molar ratio of 1.4:1 (Al:F) is sufficient to achieve effective fluoride complexation and significantly enhanced microbial activity (Sicupira et al., 2011). According to the literature, the addition of aluminum to the bioleaching setup with metal hydroxide sludge was tested. Based on preliminary tests, the optimal Al:F ratio was investigated. This was conducted under the assumption that the fluoride contained in the metal hydroxide sludge would be fully mobilized during the process. The sludge also contains ferric iron, which can complex fluoride (Rodrigues et al., 2016, 2019; Ma et al., 2017; Fritze and Hedrich, 2026). Therefore, the optimal aluminum addition had to be determined in parallel to the bioleaching tests using a ratio of 1.5:1 Al:F (see section 3.2). It was essential to achieve optimal microbial activity without introducing more aluminum than necessary in terms of applicability.

**Figure 1:**
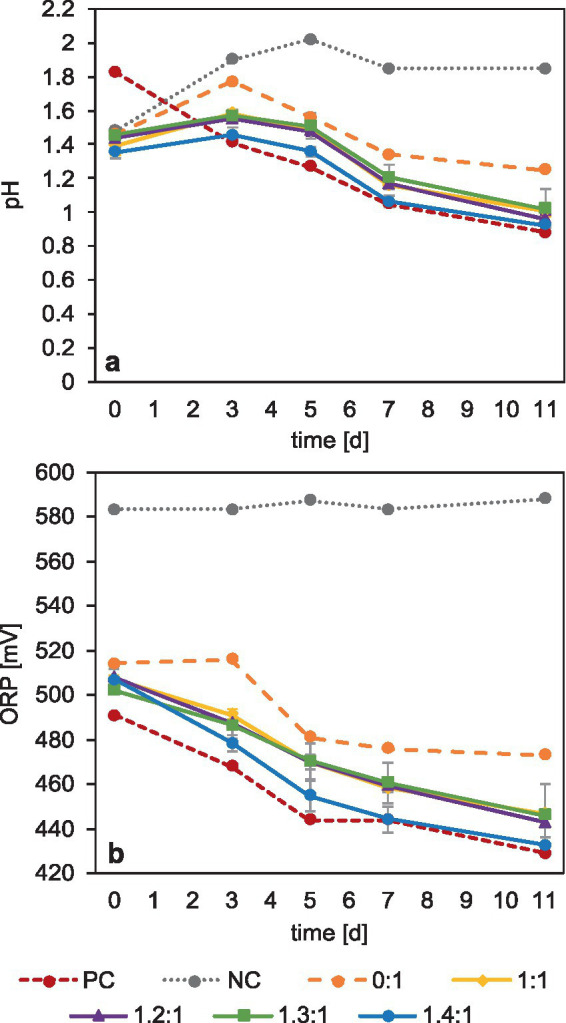
Mean and standard deviation (error bars) of pH **(a)** and redox potential (ORP vs. Ag/AgCl) **(b)** during cultivation of *At. thiooxidans* on 3% (w/v) metal hydroxide sludge and 1% sulfur as substrate in shake flasks with different Al:F ratios; PC, positive control (cultivation in the absence of metal hydroxide sludge); NC, negative control (without inoculum and 1.4:1 Al:F ratio).

Figure 1 shows the monitoring of the pH and redox potential during growth tests at different Al:F ratios in the presence of 3% (w/v) metal hydroxide sludge. Without the addition of aluminum (0:1), a pH increase of up to 1.8 was observed on day 3, followed by a decrease to 1.3 on day 11. The development of pH values shows that there was already a delay or inhibition of microbial sulfur oxidation by day 3. The pronounced initial increase in pH values can be attributed to the dissolution of the sludge in the acidic medium. This suggests limited microbial sulfur oxidation activity at this stage to counteract the effect. Only a slight decrease in redox potential was observed, which was related to the slight decrease of the pH value. For the Al:F ratios of 1:1, 1.2:1 and 1.3:1, a similar behavior was observed. The pH value dropped to 1.2 by day 7 and reached 1.0 by day 11. The redox potential shows a decrease from approx. 500 mV to 450 mV on day 11. Since the reduction is driven by metabolites produced during sulfur oxidation (Breuker and Schippers, 2024), a decreasing pH is coupled to the reduction of ferric iron and consequently a lowering of the redox potential. Most active microbial sulfur oxidation was observed at an Al:F ratio of 1.4:1, which is in accordance with the study of Sicupira et al. (2011). By day 7, the pH had already dropped to 1.0, and the redox potential had reached 440 mV. The most notable increase in ferrous iron concentration (see Supplementary Figure S1) was observed when 1.4:1 Al:F was added, whereas it was noticeably lower in the sample without Al addition. For subsequent bioleaching experiments, an Al:F ratio of 1.4:1 was assumed to maintain optimum conditions for microbial activity. Notably, the experiments also showed sufficient microbial activity at lower ratios. Fluoride inhibition is likely most critical at the beginning of bioleaching, when fluoride is rapidly dissolved and immediately affects microbial activity. Fluoride can be complexed by ferric iron (Rodrigues et al., 2016, 2019; Ma et al., 2017); however, the F:Fe ratio remains too low (approximately 1:4), even after complete dissolution of ferric iron from the sludge, whereas a ratio of about 1:10 would be required (Ma et al., 2017). Therefore, the addition of aluminum at a ratio of 1.4:1 Al:F was efficient to achieve high bioleaching activity with the sludge (Fritze and Hedrich, 2024). The positive effect of aluminum on the bioleaching of fluoride-containing copper sulfide ores was already demonstrated by Veloso et al. (2012). At least 80% copper extraction was achieved by minimizing the fluoride inhibition at an Al:F ratio of 2:1. Fluoride toxicity was also mitigated by adding Al in a ratio of 1.4 Al:F during bioleaching of a fluoride-containing, low-grade copper ore with *Sulfobacillus thermosulfidooxidans* (Sicupira et al., 2011).

### 3.2 Bioleaching in shake flasks

**Figure 2:**
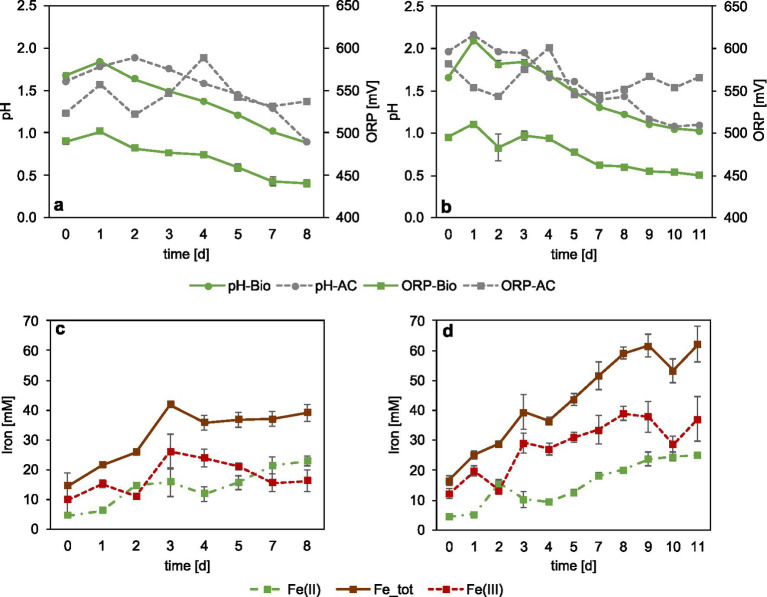
Mean and standard deviation (error bars) of pH, ORP (vs. Ag/AgCl), and iron-concentration in solution during aerobic reductive bioleaching of 3% (w/v) **(a,c)** and 5% (w/v) **(b,d)** metal hydroxide sludge with *At. thiooxidans* at 30 °C; AC, abiotic control (post-acidification with 2 M H_2_SO_4_); Fe_tot, total iron concentration; addition of Al as 1.5:1 Al:F ratio.

Figure 2 presents the results obtained from aerobic reductive bioleaching experiments conducted in shake flasks using metal hydroxide sludge at solid loads of 5% (w/v) and 3% (w/v). Aluminum was added to counteract the inhibitory effect of fluoride.

For the experimental setup with 3% (w/v) solid content, a clear decrease in pH from 1.8 on day 4 to 0.89 on day 8 was observed. The strong decrease in pH indicates a high level of microbial sulfur oxidation activity, supporting aerobic ferric iron reduction. The redox potential decreased from 500 mV to 474 mV on day 4 and finally to 440 mV on day 8. The total iron concentration increased until day 5, where most of the ferric iron from the sludge was dissolved with 57% ferric iron and 43% ferrous iron. After 7 days 41% ferric iron and 59% ferrous iron were present indicating ferric iron reduction by sulfur intermediates, produced during microbial sulfur oxidation. Up to day 8, no noticeable increase in the ferrous iron concentration was observed.

In the setup containing 5% (w/v) sludge, a decline in pH was observed indicating active microbial sulfur oxidation. As the pH initially increased to 2.2 at the beginning of the experiment, sulfuric acid was added to adjust the pH to 1.8. Up to day 7 the pH decreased to 1.3 and reached 1.1 on day 11. Compared to 3% (w/v) solid load, the pH decrease in the 5% (w/v) bioleaching setup occurred more slowly. This could be attributed to the higher solid load and the therefore higher acid buffering capacity. The redox potential also showed a slower decrease. A redox potential of 511 mV was measured on day 1, which decreased to 450 mV by day 11. The total iron concentration increased up to day 8, indicating that the sludge dissolved until that point. An increase in the ferrous iron concentration by day 4 was measured. By the end of the experiment, 60% ferric iron and 40%, ferrous iron were still present in the leachate.

An increase in solids content led to decreased ferric iron reduction, which was notably lower compared to values reported in the literature. For instance, Marrero et al. (2015) reported 72% ferric iron reduction after 11 days of aerobic reductive bioleaching of laterite tailings with *At. thiooxidans* at 10% solid load. Similarly, Stanković et al. (2022) observed up to 100 mM total iron, with 70% as ferrous iron, after 21 days of bioleaching of limonitic laterites using *At. thiooxidans*, indicating more efficient ferric iron reduction under comparable or even higher iron concentrations. Nevertheless, a direct comparison of the various reductive bioleaching studies is difficult, as ferric iron reduction is affected by multiple factors, including the different mineralogical composition of the laterite ores (Hetz and Schippers, 2025), experiment duration time and the presence of dissolved substances in the leachate. These vary depending on the type of material bioleached, which may inhibit microbial activity and thereby reduce bioleaching efficiency. Moreover, the experimental duration of these tests was comparatively short, which may have limited the extent of iron reduction. Therefore, process optimization should aim to maximize ferric iron reduction. In a recent study, Hetz and Schippers (2024) examined aerobic reductive bioleaching of laterites using pure and mixed cultures of *At. thiooxidans*. After 15 days, most of the total iron (approximately 30–50 mM) was present in the reduced form (Fe^2+^), accounting for 99–100%, both with and without pH control. This demonstrates that complete ferric iron reduction is achievable.

**Figure 3:**
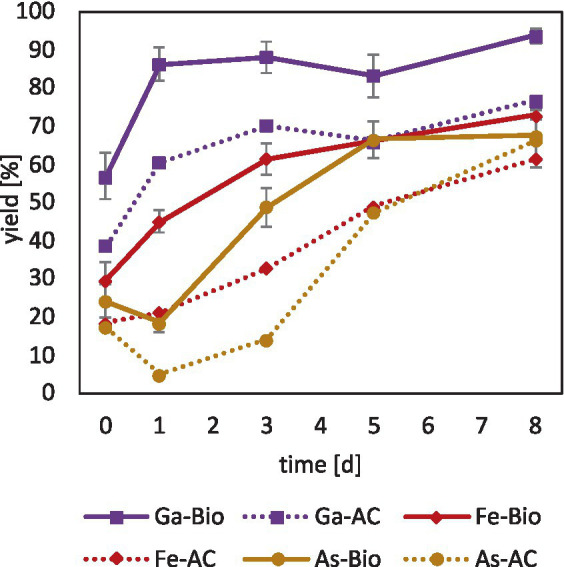
Mean and standard deviation (error bars) of element leaching yield for aerobic reductive bioleaching shake flask experiments with 3% (w/v) metal hydroxide sludge using *At. thiooxidans*; Bio, biotic approaches; AC, abiotic controls (pH adjustment by acid addition analogously to the pH values of the biotic approaches).

Iron in the metal hydroxide sludge occurs mainly as amorphous ferrihydrite (Haubrich et al., 2020). The dissolution of ferrihydrite by microbially-produced sulfuric acid as well as via reductive bioleaching is expected to result in the release of Fe, As and Ga. The yields of Ga, As and Fe from the bioleaching experiments using 3% (w/v) metal hydroxide sludge are presented in Figure 3. The dissolution of metals over the course of the experiment shows that Ga dissolves quite rapidly within the first day (86%) and finally reached 94% Ga in solution. This may be attributed to the initially low pH value, that is needed for the acidophilic microorganisms. In previous studies on metal hydroxide sludge, Haubrich et al. (2020) observed that gallium is almost completely dissolved at pH of ≤ 2.0, during chemical leaching with sulfuric acid. Bioleaching is less effective for Ga dissolution, as Ga is primarily dissolved due to the low pH of the medium. The yields for As demonstrate that its dissolution does not proceed at a constant rate from the beginning of the experiment. An increase in arsenic concentration from 24% on day 1 to 68% on day 8 was observed. That confirms the assumption that As is released during ferrihydrite dissolution. The observed iron yield of 73% suggests that iron was not completely dissolved, and that a portion may have been bound by the released silicic acid. The abiotic controls showed lower yields compared to the biotic setups with 77% Ga, 66% As and 62% Fe. It can be hypothesized that the leaching of the metal hydroxide sludge is facilitated by microbial activity. However, it should be noted that the abiotic controls were pH-adjusted based on the biotic setups. Furthermore, the abiotic experiments represent simplified controls, and therefore, these findings need to be confirmed through replicate experiments. For the reductive bioleaching, similar values for the yield of gallium were observed at 5% (w/v) solid content (see Supplementary Figure S2). To summarize, the biotic setups show good microbial leaching activity for both 3% (w/v) and 5% (w/v) solid content. However, in the 5% (w/v) setup, a longer cultivation period is required to observe ferric iron reduction. This may be due to the higher solid content, which typically leads to higher acid buffering capacity, toxicity and reduced mixing efficiency. In particular, limited oxygen transfer can restrict microbial oxidation processes. The application of bioleaching for this metal hydroxide sludge should focus on reducing as much ferric iron as possible in the leaching solution in order to allow an efficient downstream process with only ferrous iron in solution. Therefore, it was decided to use a solid load of 3% (w/v) for further optimization in bioreactors at larger scale.

### 3.3 Optimizing bioleaching in bioreactors

Based on the shake flask experiments, ferric iron reduction was found to be more effective at a solid load of 3% (w/v) metal hydroxide sludge. Additionally, an optimal Al:F molar ratio of 1.4:1 was identified. Two bioreactor runs were conducted, employing either stepwise (Bosecker, 1987) or direct addition of the sludge. In the stepwise approach, 1% (w/v) of the sludge was added incrementally until the total solid load was reached. Initially, 1% (w/v) was added, and further additions were made when the pH dropped below 1.3–1.2. This strategy was based on findings by Johnson et al. (2017), who showed that aerobic ferric iron reduction by *At. thiooxidans* occurred at pH values of 1.2 and lower.

**Figure 4:**
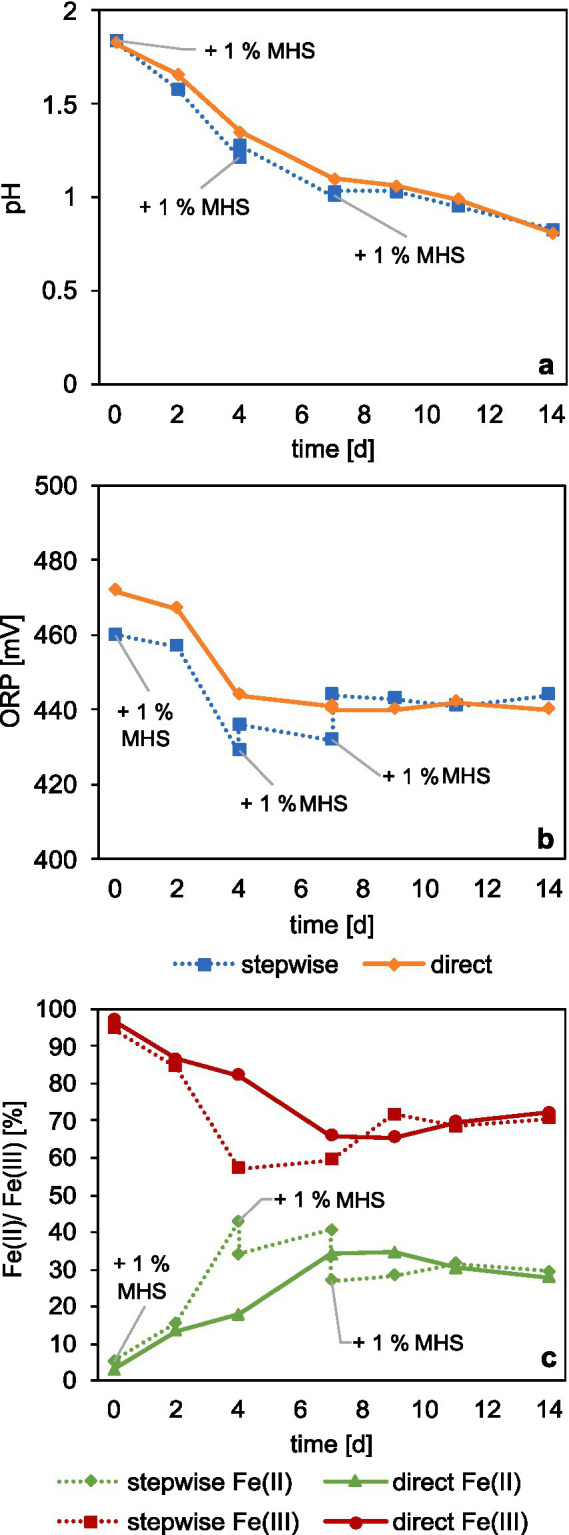
Monitoring of pH **(a)**, redox potential (ORP vs. Ag/AgCl) **(b)**, and Fe(II)/Fe(III) ratio **(c)** during aerobic reductive bioleaching in 2 L-stirred tank bioreactor (1 L working volume) at 3% (w/v) metal hydroxide sludge with stepwise and direct addition of the sludge; MHS, metal hydroxide sludge.

Figure 4 presents the monitoring of pH, redox potential as well as the iron concentrations in solution for both bioreactor experiments. Both experiments show a clear drop in pH, indicating a high level of microbial sulfur oxidation activity. The bioleaching test with the complete initial addition of the sludge reached a pH value of 1.1 by day 7, whereas the stepwise sludge addition reached a pH value of 1.0. At the end of the experiments, the pH value decreased to approximately 0.8. With stepwise addition of the sludge, the redox potential decreased from 460 mV to 429 mV by day 4, and reached 444 mV by day 14. During the test with initial complete sludge addition, the redox potential declined from 472 mV to 444 mV on day 7 and further to 440 mV by day 14. This drop in redox potential could be explained by the reduction of ferric iron to ferrous iron (Marrero et al., 2015; Santos et al., 2020; Stanković et al., 2022). During stepwise addition of the sludge, the temporary increase in pH values and redox potential could be explained by the alkaline nature of the freshly added material. The ferrous iron concentration increased in both experiments, which also confirms the decrease in the redox potential. In the stepwise addition experiment, 42% of the dissolved iron by day 4 was ferrous iron. Following subsequent sludge addition, the ferrous iron reached 40% by day 7. After the final sludge addition, the ferrous iron concentration decreased to 29%. As more sludge was added, the total iron concentration increased accordingly, resulting in a relative decrease in ferrous ion. The ferrous iron concentration during initial complete addition of sludge showed an increase in ferrous iron concentration to 28% at the end of the experiment. It should be noted that the sludge had been completely dissolved by day 7 (see Supplementary Figure S3), leading to an increase in the total iron concentration. For both bioreactors runs, the ferrous to ferric iron ratio remained stable from day 11 onward, indicating that no further reduction of ferric iron occurred. Comparing the bioreactor bioleaching experiments, no clear differences in ferric iron reduction was noted. The main advantage of the stepwise addition approach is that no additional sulfuric acid is required for pH control as previously described (Bosecker, 1987). From the outset, the acidic inoculum effectively prevented an increase in pH above 1.8. In a larger-scale application, this can lower the use of chemicals.

However, compared to the shake flask experiments using 3% (w/v) sludge, ferric iron reduction was less efficient. While 59% of the ferric iron was reduced after 7 days in the shake flask experiments, only 30% was reduced after 14 days in the bioreactor. It is noteworthy that both, the redox potential and the ferric iron concentration, decreased up to day 7, while the pH value remained above 1.0 throughout this period. Afterwards the pH continued to decline to a value of 0.8, but neither the redox potential nor the ferrous iron concentration showed clear changes. *At. thiooxidans* is known to survive in solutions with pH as low as 0.5 (Kelly and Wood, 2000). Nevertheless, the growth optimum for *At. thiooxidans* is pH 2.0–3.0 (Kelly and Wood, 2000). Therefore, a low pH has negative effects on cells and can reduce activity.

Furthermore, the dissolution of iron oxide minerals such as goethite during reductive bioleaching by sulfur-oxidizing acidophiles is strongly pH-dependent and can be explained by the following reactions. Protons generated during microbial sulfur oxidation promote protonation of the mineral surface and facilitate dissociation of Fe–O bonds, which is required for iron oxide dissolution and the release of soluble ferric iron. In addition, reduced inorganic sulfur species formed during sulfur oxidation may chemically reduce ferric iron but do not initiate mineral dissolution (Stanković and Schippers, 2024; Hetz and Schippers, 2025). As the pH further decreases, ferrihydrite phases may dissolve more extensively, increasing the concentration of ferric iron in solution and masking any concurrent ferric iron reduction. In addition, very low pH values may affect sulfur metabolites such as thiosulfate or hydrogen sulfide. At low pH, H₂S is almost entirely present as an undissociated molecule, with HS^−^ and S^2−^ being suppressed (Lien et al., 2022). Thiosulfate, is unstable and decomposes under strong acidic conditions (Dinegar et al., 1951; Williamson and Rimstidt, 1993). Accordingly, the potential for ferric iron reduction by sulfur metabolites probably decreases as the pH declines. Moreover, it should be considered that the fluoride present in the sludge may also affect microbial activity. As reported in the literature, the concentration of free fluoride ions increases with rising pH (Brierley and Kuhn, 2010; Li et al., 2019). According to Li et al. (2019), at pH values below 2.0, more than 95% of fluoride is present as undissociated hydrogen fluoride (HF). Furthermore, Denham and Millings (2003) indicate that at pH 1.5, fluoride is expected to exist almost entirely in the form of HF. In the bioreactor experiments, the pH was below 1.0 by day 7 and the ferric iron reduction stagnated at this point. In comparison, in the shake flasks experiment with 3% (w/v) sludge, the pH remained above 1.0 until day 7, at which ferric iron reduction was visible. For the 5% (w/v) approach, the pH was above 1.0 until day 11 (see Figure 2), when ferric iron reduction was measured. In previous studies a constant pH was set during aerobic reductive bioleaching, as, e.g., by Stanković et al. (2022) with pH set to 1.0. In the study by Hetz and Schippers (2024) investigations were carried out with pH control and resulted in a significantly higher ferric iron reduction.

In conclusion, pH appears to be a key factor for the aerobic reductive bioleaching of metal hydroxide sludge. As ferric iron reduction seems to decrease at very low pH (<1.0), a staged addition of the metal hydroxide sludge was employed to the bioleaching system, as its alkaline properties naturally counteract the acidic conditions upon dissolution.

**Figure 5:**
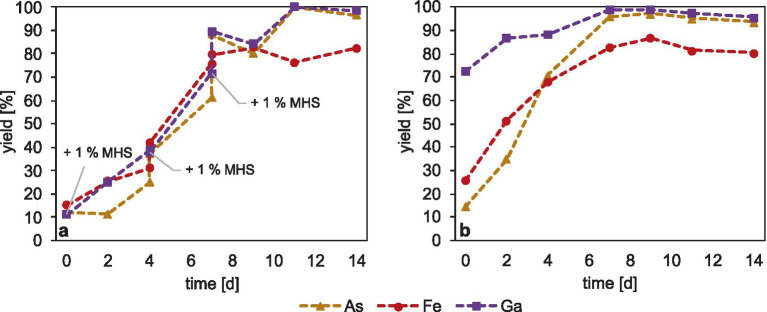
Element leaching yield during aerobic reductive bioleaching in 2 L-stirred tank bioreactors (1 L working volume) at 3% (w/v) metal hydroxide sludge with **(a)** stepwise and **(b)** direct addition of the sludge. Metal hydroxide sludge (MHS).

Figure 5 shows that the elements Ga, As, and Fe were already leached straight after the sludge was added to the process. This confirms again that the metal hydroxide sludge dissolves mainly due to the very acidic conditions. While during stepwise sludge addition (Figure 5A) iron leaching stops at day 7, Ga and As continue to leach completely until day 11. Analyses of residues from previous bioreactor experiments (data not shown) revealed SiO₂-rich solids, with pore waters containing Fe and As that formed sulfates upon drying of the solids, which may explain the incomplete recovery of iron. For the bioreactor run with direct addition of the sludge (Figure 5B), there is an increase in the yield for iron up to 80% on day 7. Ga is already 70% dissolved at the start of the experiment due to the acidic conditions and continues to increase until day 7, when it reaches almost 100%. Arsenic shows a steeper increase from 14 to 99% yield on day 7. This indicates that its release appears to be more dependent on the dissolution of iron. Based on the yields, the direct sludge addition approach seems to be more efficient, as it shows complete leaching of Ga and As after just 7 days, compared to the approach with stepwise addition, which requires 11 days for complete Ga and As leaching.

**Figure 6:**
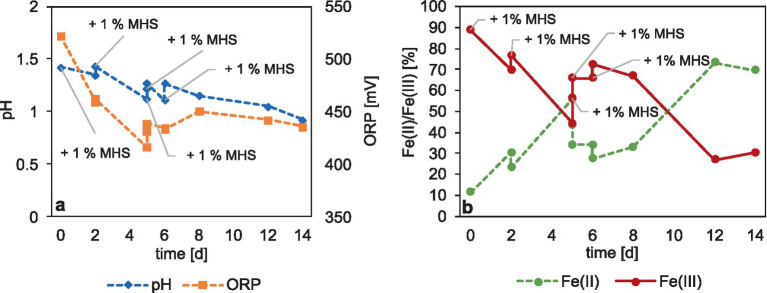
Monitoring of pH, redox potential (ORP vs. Ag/AgCl) **(a)** and Fe(II)/Fe(III) ratio **(b)** during aerobic reductive bioleaching in 2 L-stirred tank bioreactor (1 L working volume) at 5% (w/v) metal hydroxide sludge with stepwise addition of the sludge; MHS = addition of 1% (w/v) of metal hydroxide sludge.

In a subsequent bioreactor experiment, a higher solid load of 5% (w/v) was tested, involving the gradual addition of sludge to control the pH. The development of the pH and redox potential as well as the Fe(II)/ Fe(III) ratio is presented in Figure 6. After inoculation with the bacterial culture, the bioreactor pH was 1.4 and decreased steadily indicating microbial sulfur oxidation activity. After each addition of 1% (w/v) sludge the pH values increased immediately and dropped consecutively below ≤ 1.3.

The entire 5% (w/v) sludge was fed stepwise into the process up to the day 6 and the pH continued to decrease to 0.9 on day 14. The redox potential showed an initially sharp decrease, followed by a slight decline from day 6 onwards. On day 5, the Fe(II)/Fe(III) ratio increased, which correlated with the sharp drop in pH and redox potential. After 10 days the ferrous iron concentration was higher than the ferric iron one in the solution, remaining stable until day 14 without markable further increase. Reductive bioleaching at higher solid loads achieved greater ferric iron reduction than bioreactor experiments at lower solid loads, again highlighting the strong dependence of pH on ferric iron reduction during bioleaching. While only 30% ferrous iron was produced in the bioreactor run with 3% (w/v) of sludge, the gradual addition of up to 5% (w/v) succeeded in generating 70% ferrous iron in the leaching solution. From day 12 onwards, the pH dropped below 1.0, resulting in no further changes in ferrous iron concentration until the end of the experiment. Final yields of 83% for Ga, 88% for As, and 72% for Fe were obtained. Thus, for future process optimization, the sludge could be added gradually for the microbially mediated iron reduction in the leaching solution. It seems to be particularly important to control the pH and to keep it above pH 1.0 since at lower values, the process could become inefficient. In contrast, bioleaching of laterite ores with a mixed culture of six *At. thiooxidans* strains allowed for microbially mediated ferric iron reduction coupled to aerobic sulfur oxidation clearly below pH 1.0 [shown in Figures 1b, 2d; Hetz and Schippers (2025)]. Another previous study showed that ferric iron reduction with different pure cultures of *At. thiooxidans* strains occurred at pH 0.8 to 2.3 (Breuker and Schippers, 2024). Overall, this study showed that bioleaching is suited for the recycling of the metal hydroxide sludge. However, further optimization should be performed to reduce as much of the ferric iron in the leaching solution as possible to allow efficient downstream processing for the Ga recycling.

### 3.4 Implications for gallium recovery and downstream processing: bioleaching vs. hydrometallurgy

Detailed studies on recycling of metal hydroxide sludge were carried out within the frame of the national research project EcoGaIN (Haubrich et al., 2020). Acidic and alkaline leaching approaches were applied to extract the gallium. During alkaline leaching with NaOH, complete dissolution of silicic acid, along with As and Ga, was achieved, while iron remained in the solid phase as iron hydroxide. The formation of gelatinous silicic acid, which also accumulates Ga and As, hindered further separation of these metals. Acid leaching with HCl led to complete dissolution of the sludge, but also produced vast amounts of gelatinous silicic acid. Selective leaching using citric acid resulted in incomplete leaching of Fe, Ga, and As. Leaching with 1 M H_2_SO_4_ achieved complete dissolution of Fe, As, and Ga. The resulting silica was proposed to be removed by centrifugation or filtration after adding a flocculant (Haubrich et al., 2020). The study showed that Ga only dissolves significantly when iron hydroxide starts to dissolve at pH < 5.0, with Ga being almost completely dissolved at pH 2.0 and below. Up to a pH value of 3.5, higher consumption of H_2_SO_4_ was observed due to presence of calcium hydroxide/calcium carbonate, exhibiting buffering effects. At pH 1.0, Ga, As, and Fe should be completely dissolved. The optimum solid:liquid ratio for leaching with 1 M H_2_SO_4_ was determined as 1:4 (Haubrich et al., 2020). When comparing the bioleaching approach reported in this study with the above-mentioned acid leaching process within the EcoGAIN project, the hydrometallurgical process exhibited greater efficiency in terms of processing time, particularly with respect to gallium recovery. The application of reductive bioleaching for ferric iron reduction in the leaching solution requires more than 1 week. In contrast, chemical reduction required only a few hours to several days. A substantially higher solid load (25% w/v) can be processed via hydrometallurgical process, whereas the tested bioleaching approach was limited to a solid concentration of only 3–5% (w/v). In addition, biohydrometallurgical processes require considerably longer leaching times, which is a general disadvantage of biohydrometallurgy (Kumar and Yaashikaa, 2020). Nevertheless, bioleaching also offers several advantages. Notably, a clear reduction in sulfuric acid consumption was observed, as *At. thiooxidans* produces sulfuric acid autonomously by oxidizing elemental sulfur as a substrate. Bioleaching has as also a reduced energy demand due to moderate process temperatures (in this case 30 °C) (Stanković et al., 2022). Furthermore, at pH 1.0, the concentration of sulfuric acid corresponds to approximately 0.05 M H₂SO₄. Despite the low acid concentration in relation to the solid load, near-complete dissolution of Ga and As was achieved. This may be attributed partly to the extended process time. Additionally, microbial activity continuously produces sulfuric acid to sustain proton generation, which promotes leaching. The buffering effect observed in the hydrometallurgical process was also evident in this study. During direct addition of the metal hydroxide sludge, subsequent acidification was necessary to maintain optimal conditions for the microorganisms. This issue was reduced by the stepwise addition of sludge to the bioleaching process. Due to the continuous production of sulfuric acid by the microorganisms, they actively counteract the buffering capacity of the material throughout the leaching process. After successful leaching of the sludge using mineral sulfuric acid or bioleaching, the silicic acid needs to be removed before further processing of the leaching solution. As previously described by Haubrich et al. (2020), ferric iron needs to be completely reduced to ferrous iron at pH values of 1–2. During the hydrometallurgical process development, this was achieved by adding metallic iron such as Fe powder or reinforcing steel to the leach solution. Subsequently, the Ga could be precipitated as poorly soluble GaAsO_4_/Ga-hydroxide by adjusting the pH. At pH 4.5–5, the maximum GaAsO_4_/Ga-hydroxide precipitation was reached (Haubrich et al., 2020). Reductive bioleaching provides the benefit of enabling ferric iron reduction directly during the leaching process, thereby minimizing or even eliminating the requirement for additional chemical ferric iron reduction. Incomplete ferric iron reduction may lead to the precipitation of ferric iron phases during Ga precipitation at pH 4–4.5. Since previous preliminary bioleaching experiments did not achieve complete ferric iron reduction, the remaining ferric iron in the solution needed to be further reduced using elemental iron (Fe^0^). Subsequently, the Ga can be quantitatively precipitated. The addition of Al may interfere with Ga precipitation due to potential co-precipitation effects, which should be investigated in more detail in future process optimization studies. The solution remaining after the Ga separation is a waste product, which requires further treatment. Arsenic can be removed by partially re-oxidizing ferrous iron using H₂O₂, thereby forming ferric iron phases that precipitate at pH > 3.3 and quantitatively adsorb arsenic. The remaining solution still contains ferrous iron which can be oxidized to ferric iron for consecutive schwertmannite formation. Iron could also be extracted and used as Fe powder, e.g., for 3D printing (Haubrich et al., 2020; Tirado-González et al., 2024). A high SiO_2_ (70–75%) content was observed in the residues from the previous bioleaching tests (data not shown). SiO_2_ could be further converted to waterglass (Na_2_SiO_3_) (Wiemer and Hesse, 1989; Haubrich et al., 2020), by adding NaOH in a stoichiometric amount. Amorphous SiO_2_ could also be used as an activator to produce geopolymers (Haubrich et al., 2020; Matinfar and Nychka, 2023).

## 4 Conclusion

During the production of GaAs semiconductors, a significant amount of gallium is lost as waste, including Ga-containing metal hydroxide sludge from neutralization processes (Haubrich et al., 2020). This study investigates the potential of aerobic reductive bioleaching for recycling metal hydroxide sludge, building on previous hydrometallurgical studies within the frame of the German EcoGAIN project. The bioleaching process was conducted using *At. thiooxidans*, which is capable of reducing ferric iron under aerobic conditions while producing sulfuric acid. Initial results indicated that fluoride in the sludge inhibited bacterial activity, which was mitigated by adding minor amounts of aluminum to the process with an Al:F ratio of 1.4:1. Shake flask experiments showed that ferrous iron represented 60 and 40% of the total iron in the leachate after processing sludge concentrations of 3 and 5% (w/v), respectively. In bioreactor experiments with 3% (w/v) metal hydroxide sludge, only 30% of the iron was reduced after 14 days. This outcome indicates a clear pH dependence of the iron reduction, which appeared to plateau at pH values below 1.0. A subsequent bioreactor experiment was conducted with a gradual increase in sludge concentration up to 5% (w/v). This approach maintained the pH above 1.0, resulting in a 70% reduction of iron after 14 days. For 3% (w/v) sludge, metal recoveries were 80% Fe, 100% Ga, and 99% As. This study demonstrates that aerobic reductive bioleaching is a possible route for recycling Ga-containing metal hydroxide sludge, though further optimization is required to reduce iron levels in the leachate for improved gallium recovery.

## Data Availability

The raw data supporting the conclusions of this article will be made available by the authors, without undue reservation.
