# Supplementary material for: Bioleaching of GaAs metal hydroxide sludge: a biohydrometallurgical alternative to conventional acid leaching
Source: Front Microbiol. 2026 Jun 10;17:1807986. doi: 10.3389/fmicb.2026.1807986 (PMC13290815; doi:10.3389/fmicb.2026.1807986)
Supplement: Supplementary file 1 [file Supplementary_file_1.docx]

Supplementary Material

Bioleaching of GaAs metal hydroxide sludge: a biohydrometallurgical alternative to conventional acid leaching

Mareike Thea Fritze^1^, Frank Haubrich^2^, Anna Otto^1^ Axel Schippers^3^, Sabrina Hedrich^1^

^1^ Department of Biosciences, TU Bergakademie Freiberg, Freiberg, Germany

^2^ G.E.O.S. Ingenieurgesellschaft mbH, 09633 Halsbrücke, Germany

^3^ Federal Institute for Geosciences and Natural Resources (BGR), Hannover, Germany

* Corresponding Author: sabrina.hedrich@bio.tu-freiberg.de


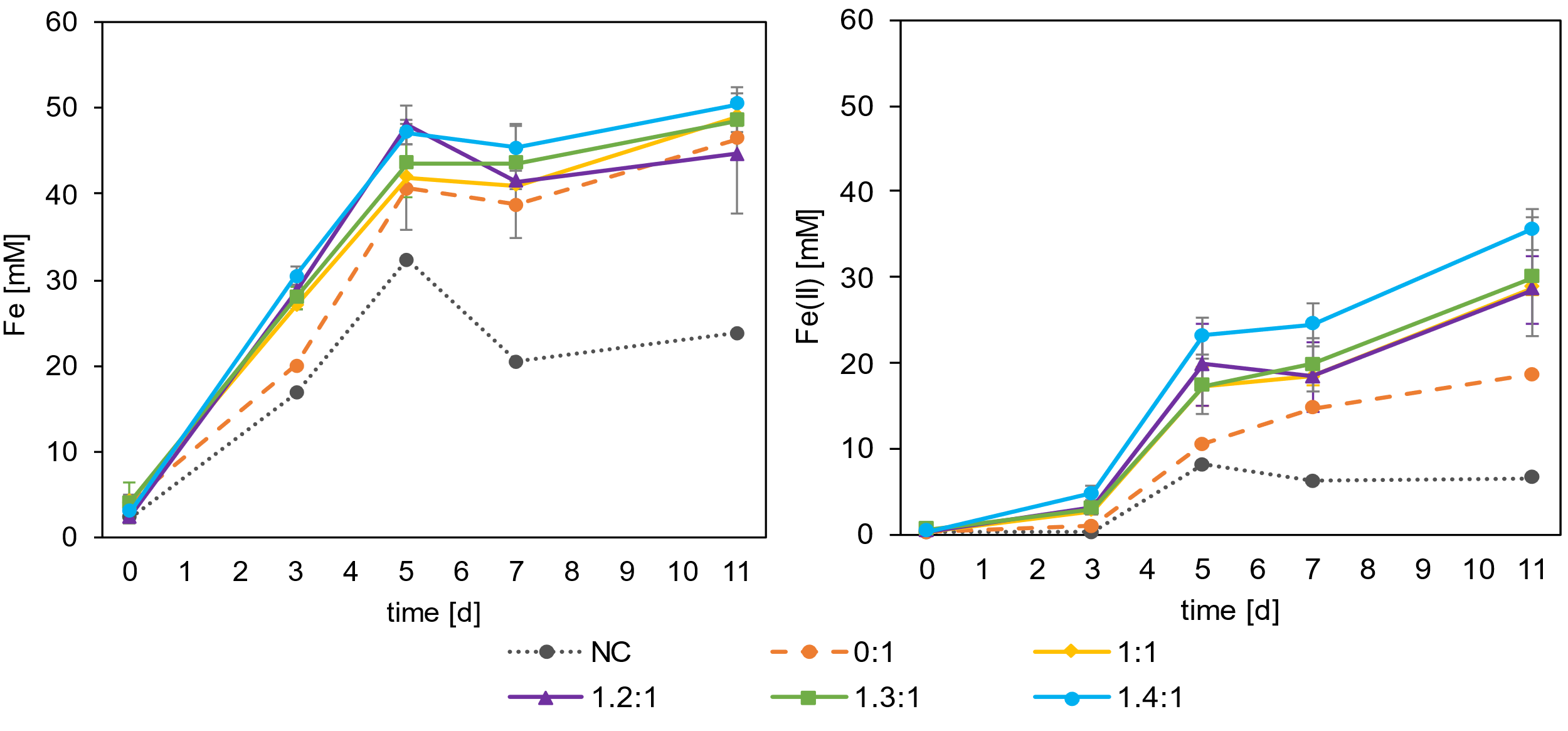


Figure S1: Mean and standard deviation (error bars) of total iron (left) and ferrous iron (right) during cultivation of *At. thiooxidans* with 3% (w/v) metal hydroxide sludge and 1% sulfur as substrate in shake flasks at different Al:F ratios; NC = negative control (without inoculum and 1.4:1 Al:F ratio)


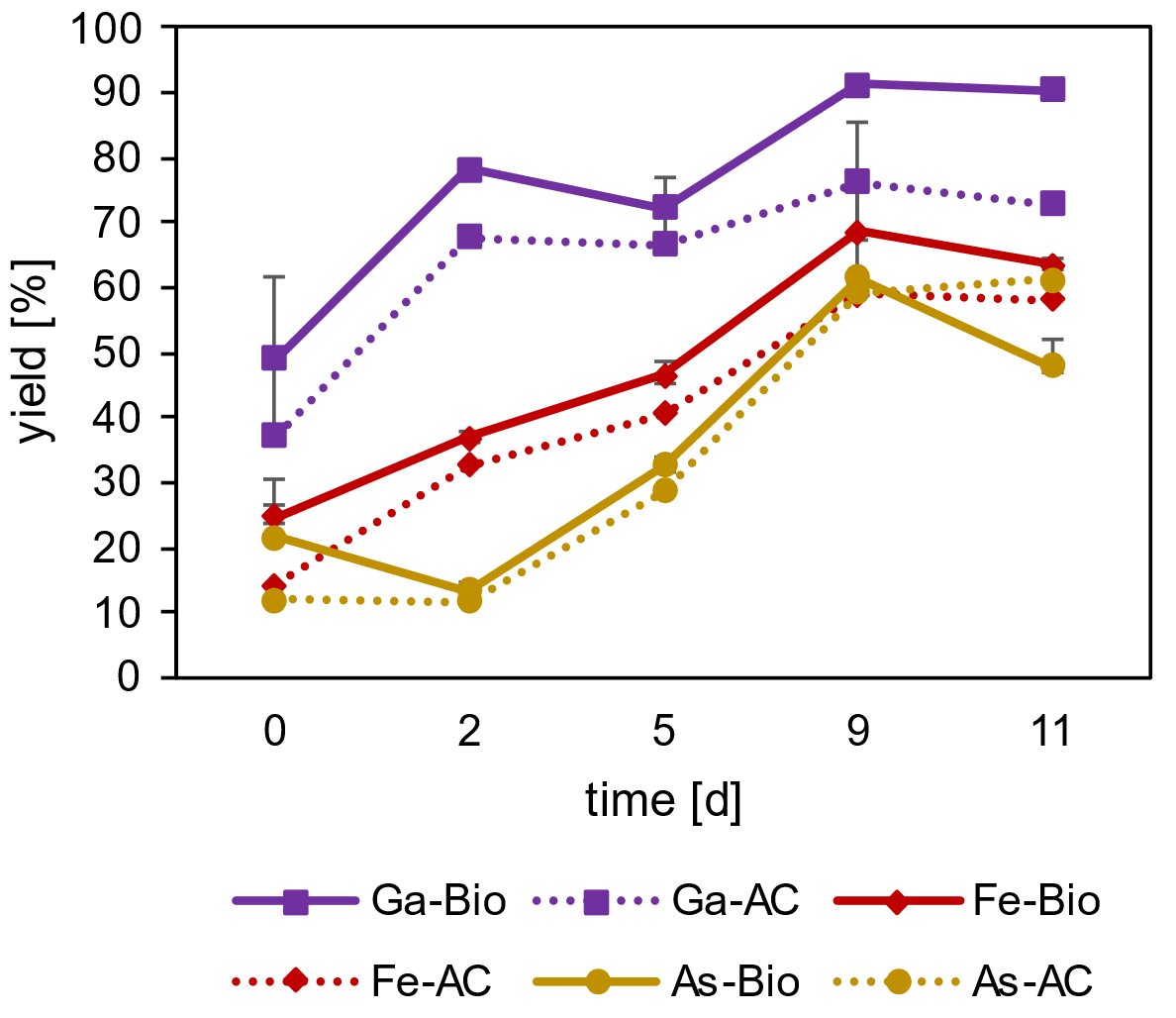


Figure S2: Mean and standard deviation (error bars) of metal(loid) yield from aerobic reductive bioleaching of 5% (w/v) metal hydroxide sludge using *At. thiooxidans*; Bio= biotic approaches; AC = abiotic controls (pH acidified analogously to the biotic assays)


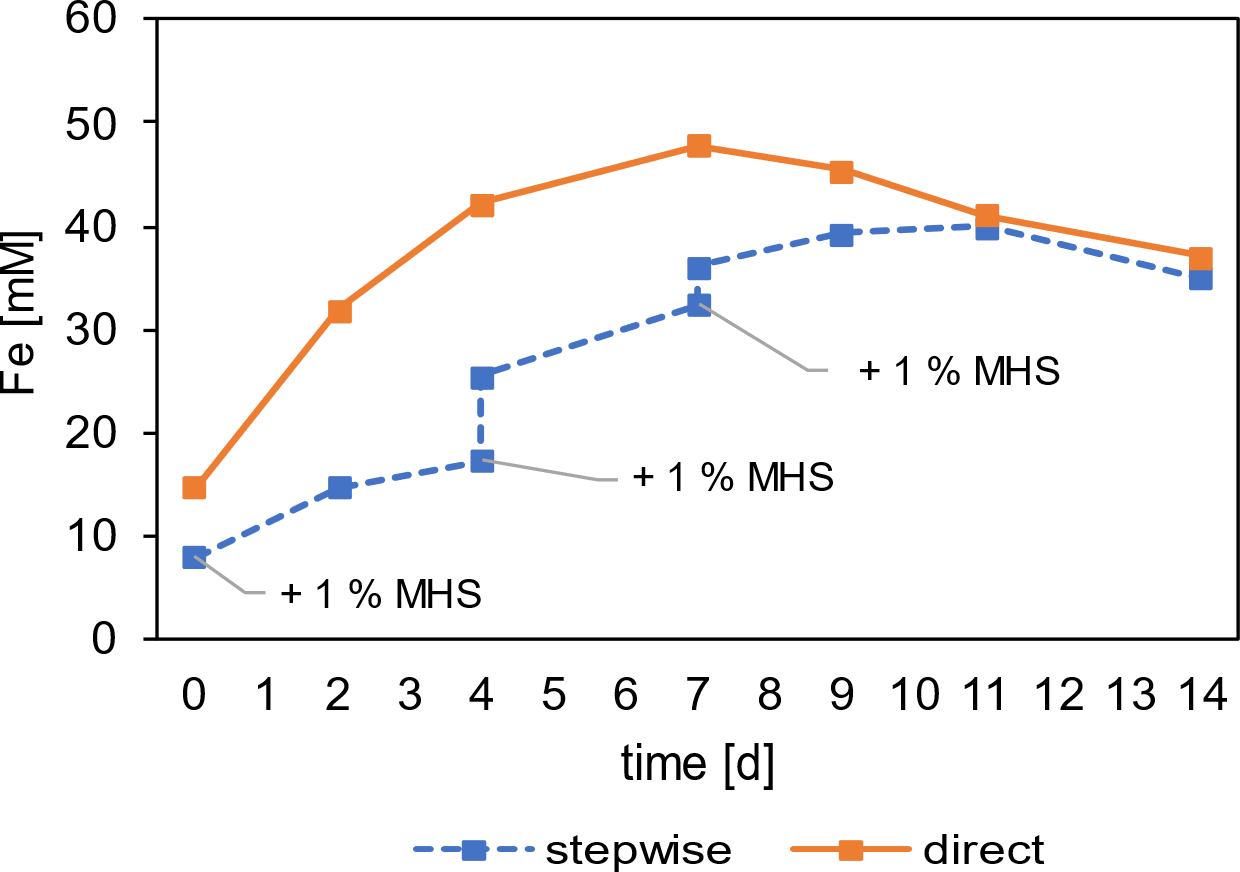


Figure S3: Monitoring of total iron concentrations during aerobic reductive bioleaching in a 2L- stirred tank bioreactor (1L working volume) with 3% (w/v) metal hydroxide sludge with stepwise or direct addition of the sludge; MHS, metal hydroxide sludge

**
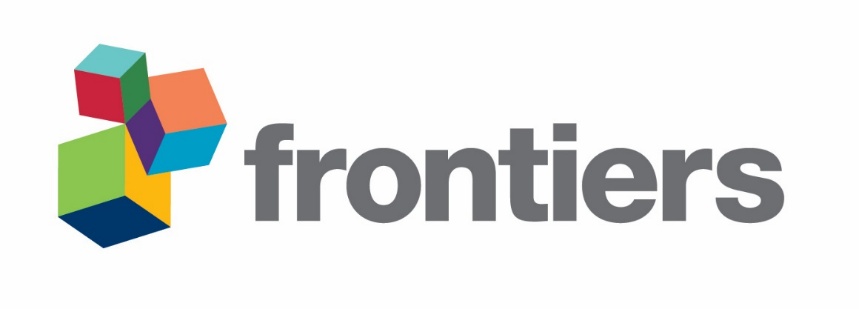
**
